# How Does Mechanical Pearling Affect Quinoa Nutrients and Saponin Contents?

**DOI:** 10.3390/plants10061133

**Published:** 2021-06-03

**Authors:** Sifeddine Rafik, Mohamed Rahmani, Juan Pablo Rodriguez, Said Andam, Amine Ezzariai, Mohamed El Gharous, Salwa Karboune, Redouane Choukr-Allah, Abdelaziz Hirich

**Affiliations:** 1 Agricultural Innovation and Technology Transfer Center (AITTC), Mohammed VI Polytechnic University (UM6P), Ben Guerir 43150, Morocco; sifeddine.rafik@um6p.ma (S.R.); mohamed.elgharous@um6p.ma (M.E.G.); 2 Department of Food and Nutritional Sciences, Section of Agricultural and Food Industries, Agronomic and Veterinary Medicine Hassan II Institute, Rabat 10112, Morocco; rahmanimohammed@yahoo.fr (M.R.); andamsaiid@gmail.com (S.A.); 3 International Center for Biosaline Agriculture, Directorate of Programs, Dubai 14660, United Arab Emirates; jprodriguezcalle@yahoo.fr; 4 African Sustainable Agriculture Research Institute (ASARI), Mohammed VI Polytechnic University (UM6P), Laayoune 70000, Morocco; Amine.Ezzariai@um6p.ma; 5 Department of Food Science and Agricultural Chemistry, McGill University, 21111 Lakeshore Road, Sainte-Anne-de-Bellevue, QC H9X 3V9, Canada; salwa.karboune@mcgill.ca; 6 Department of Horticulture, Agronomic and Veterinary Medicine Hassan II Institute, Rabat 10112, Morocco; redouane53@yahoo.fr

**Keywords:** pearling, postharvest, saponin, minerals, processing, seed bran, quinoa seeds quality, nutrition

## Abstract

Agriculture is facing many challenges, such as climate change, drought, and salinity, which call for urgent interventions for fast adaptation and crop diversification. The introduction of high-value and stress tolerant crops such as quinoa would be a judicious solution to overcome constraints related to abiotic stress and to increase land productivity and farmers’ incomes. The implementation of quinoa in Morocco has not been supported by a full valorization program to control the quality of quinoa seeds. The novelty of this work is to assess the pearling operation as an efficient method of saponins removal as well as the determination of total residual saponins. This study aimed to evaluate the effects of several pearling durations on nutrient and saponin content of quinoa seeds of three tested varieties (Puno, Titicaca, and ICBA-Q5). Five pearling durations were tested (0, 2, 4, 6, 7, and 8 min) using a locally manufactured pearling machine. The results indicated that a pearling duration of two minutes was enough to reduce total saponin content from 0.49% to 0.09% for Puno variety, from 0.37% to 0.07% for Titicaca variety, and from 0.57% to 0.1% for ICBA-Q5 variety. Our results showed that pearling slightly reduced protein, total fat, and moisture contents for all varieties except for Puno, where total fat content slightly increased with the pearling. Puno variety had the highest seed content in terms of protein and total fat; the ICBA-Q5 variety had the lowest. Titicaca had the highest bran content in terms of protein and total fat, ICBA-Q5 had the highest bran content in terms of ash and the lowest bran content in terms of protein and total fat, and Puno had the lowest bran content in terms of ash. Pearling had no significant effect on macronutrient contents in the processed seed, but it resulted in a very highly significant difference for most of them in the bran except for Mg and S. Regarding seed content in terms of micro-nutrients, statistical analysis showed significant differences between varieties in terms of Zn, Cu, and Mn contents, but no significant difference was recorded for Fe or B. Pearling had no significant effect on seed micronutrient contents. Therefore, to retain maximum nutritional content in the quinoa and maintain quinoa integrity, it is necessary to limit the pearling duration of quinoa to two minutes, which is enough to reduce saponin content below the Codex Standard threshold (0.12%).

## 1. Introduction

Water scarcity and drought are becoming direct consequences of climate change on a global scale in addition to soil salinization, especially in the Middle East and North Africa region (MENA) [1,2]. The introduction of salt-tolerant crop species is being considered as a great alternative to overcoming these environmental challenges. In this regard, quinoa (*Chenopodium quinoa* Willd.) is one of the plants with significant potential to maintain food security in countries suffering from arid conditions. Several research works have been conducted to introduce, develop, and promote quinoa crops around the world. The number of countries cultivating this staple crop has increased rapidly, from eight in 1980 to more than 120 countries in 2018 [3].

Regarding Morocco, quinoa was introduced for the first time in the Khénifra region in 1999 within the framework of the BAFI/BYU-IAV Hassan II project where 14 accessions were tested for adaptation trials [4]. Then, several research trials were carried out, especially in Agadir [5,6,7] and Rehamna [8,9,10,11], where several parameters were taken into account, such as irrigation frequencies, water quality, and soil nature. The Food and Agriculture Organization of the United Nations (FAO) recognized, in 2013, quinoa as a leading crop for food security and sustainability in the context of global change [12]; this initiative aimed to improve access and awareness of the quinoa value chain and accelerate the development of this crop around the world.

Quinoa is not only receiving attention worldwide due to its adaptability to different agro-environmental growth conditions, but also its high nutritional quality [13]. Over the last 20 years, quinoa (*Chenopodium quinoa* Willd.) has become a popular food, particularly in Europe and North America [14], because of the growing interest in vegetarian diets, its high nutritional quality, and the increase in people suffering from celiac disorders. Several studies have shown that quinoa is a very interesting food, because of its complete nutritional characteristics [15]. It is a starchy dicotyledonous seed named a pseudo-cereal. The protein content of quinoa seeds is substantial (12% to 20%) compared to corn (10%), rice (8%), and wheat (13%). In addition, a valuable characteristic of quinoa is the quality of its amino acid composition as well—especially the presence of lysine (5.1–6.4%) and methionine (0.4–1.0%). These amino acids are not abundant in vegetable diets [16,17]. Furthermore, starch is the major component of quinoa seeds and represents about 52–69.2% of the dry weight basis [18,19]; it is genuinely located in the perisperm. Quinoa is also considered a good source of fat; fat varies from 2% to 10% of quinoa depending on the variety [20]. Compared to other cereals, quinoa has high levels of calcium, phosphorus, magnesium, iron, zinc, potassium, and copper [16,21]. Some studies showed that the antioxidant properties of quinoa seeds and flour can be used as taken advantage of when used as ingredients to enrich food preparations. For instance, they can be used in meat industries that require antioxidants from natural sources to substitute synthetic antioxidants because of their negative effects [22,23].

The quality of quinoa is considered as one of the most crucial aspects that should be taken into consideration in order to justify its consumption and its manufacturing on an industrial scale. Nevertheless, quinoa quality in Morocco is facing many technical constraints, such as the bitter taste, which is a major organoleptic default. This sensorial limitation is explained by the lack of control of operations that aim to remove saponin from seeds and the dosage of residual saponin. Quinoa’s saponins are considered anti-nutritional factors that cause bitterness. Additionally, saponins may cause digestive irritation [24]. Besides, saponins’ concentration and distribution in seeds are variables depending on varieties and climatic conditions [25]. According to the quinoa standard (step 8 before publication), the Environmental Working Group (EWG) agreed to a saponin content of 0.12% as a maximum. This threshold was adopted from the Bolivian standard “Andean Standard NB 0038 for processed quinoa seeds” [26], measured through the afrosimetric method (foam test) developed by Koziol et al. [27]. However, a standardized method for total saponin quinoa dosage has not yet been officially published. The presence of saponins requires processing in order to eliminate saponins partially or totally. They are mainly present in the pericarp (86%), which explains why external abrasion is one of the best options for saponin removal [28,29]. Many methods have been developed for saponin removal; the most commonly adopted one is the mechanical abrasion process. It is an operation based on physical frictions to remove the bran. It allows obtaining a by-product (seed bran) which is rich in saponins and other nutrients. This process must eliminate saponins while preserving seed nutrients and physical properties. The optimization of the process can be done by adjusting a set of parameters involved in the mechanical abrasion operation, such as the duration of the abrasion (pearling time) and milling performance. This research focused on pearling duration as a principal factor to optimize the pearling process. The optimal pearling duration will allow saponin elimination, and preservation of the overall nutrient profile (macronutrients and micronutrients) and morphological aspects. Moreover, this research investigated the behavior of three quinoa cultivars in response to the pearling process.

## 2. Results

### 2.1. ANOVA Results

Table 1 summarizes results of the ANOVA (analysis of variance) of investigated parameters for both processed seeds and seed bran. Pearling had no significant effect on macronutrient contents in the processed seeds of any variety. It caused a very highly significant difference (*p* < 0.001) for most of the micronutrients except for Mg, S, and Fe. Pearling duration caused significant differences (*p* < 0.001) in 1000 seed weight, moisture, and saponin content for all quinoa varieties. However, there was no significant effect on the nutritional contents of quinoa seed varieties.

### 2.2. Physical Parameters

#### 2.2.1. Pearling Efficiency

**Figure 1:**
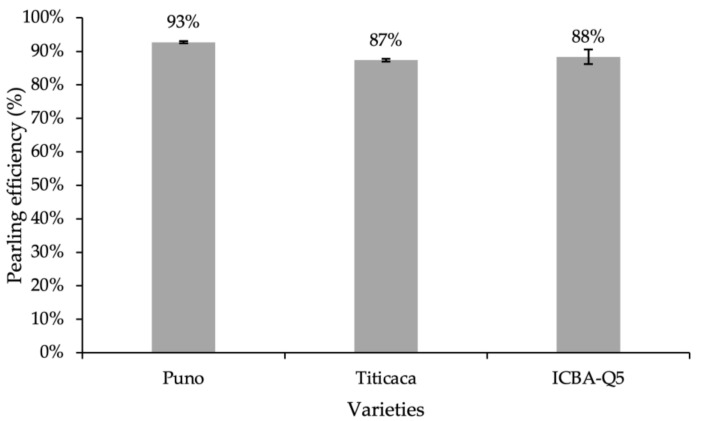
Variation of pearling efficiency per variety. Error bars indicate the standard deviation. Treatments without a common letter are significantly different at *p* < 0.05.

Pearling efficiency (PE) is defined as the ratio between processed seeds and raw seeds. Figure 1 shows the variation in PE after 8 min of pearling. Presented data indicate that pearling efficiency varies from one variety to another, and Puno variety has the highest pearling rate and Titicaca has the lowest.

#### 2.2.2. 1000 SW

**Figure 2:**
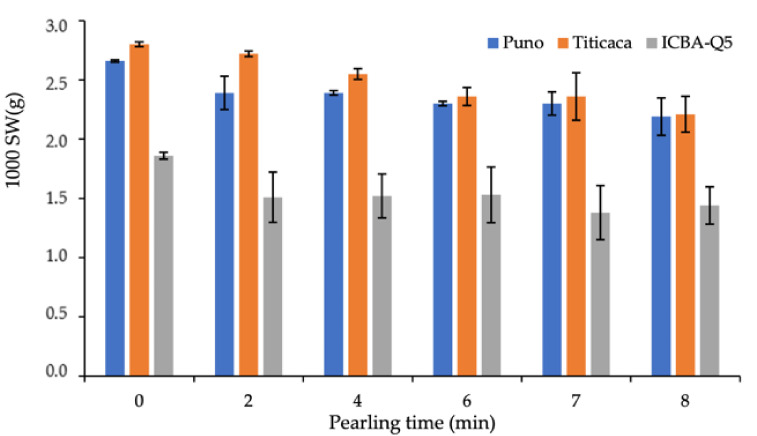
Variation of 1000 SW in response to pearling duration. Error bars indicate the standard deviation. Treatments without a common letter are significantly different at *p* < 0.05.

Figure 2 presents the variation of 1000 SW in response to pearling time. The obtained data clearly indicate that average SW decreases with the pearling as a result of seed polishing and bran removal. After 2 min of pearling, average seed weight decreased by 10, 3, and 19% for Puno, Titicaca, and ICBA-Q5 varieties, respectively. However, it decreased by 18, 21 and 23% after 8 min of pearling for Puno, Titicaca, and ICBA-Q5 varieties, respectively.

#### 2.2.3. Morphological Aspects

**Figure 3:**
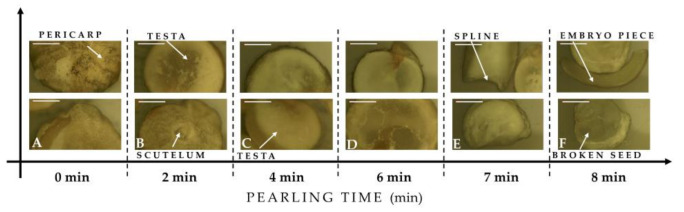
Optical microscopy images of raw (0 min) and treated seeds of Puno Variety after different pearling durations. Pearling duration: (**A**). Raw seed (0 min); (**B**) 2 min; (**C**) 4 min; (**D**) 6 min; (**E**) 7 min; (**F**) 8 min. (Bar length: 500 µm).

Figure 3 shows morphological aspects of Puno seed in response to the pearling process. The obtained pictures show the effects of mechanical friction during the abrasion. After 6 min of pearling, quinoa seeds start to have some damage, and the abrasions mill the embryo and start damaging the perisperm. After 2 min of pearling, the pericarp is entirely removed, and the seed becomes transparent.

### 2.3. Chemical Parameters

Table 2 summarizes the results of Tukey’s HSD post hoc test, indicating homogenous groups using small letters.

#### 2.3.1. Proximate Analysis

**Figure 4:**
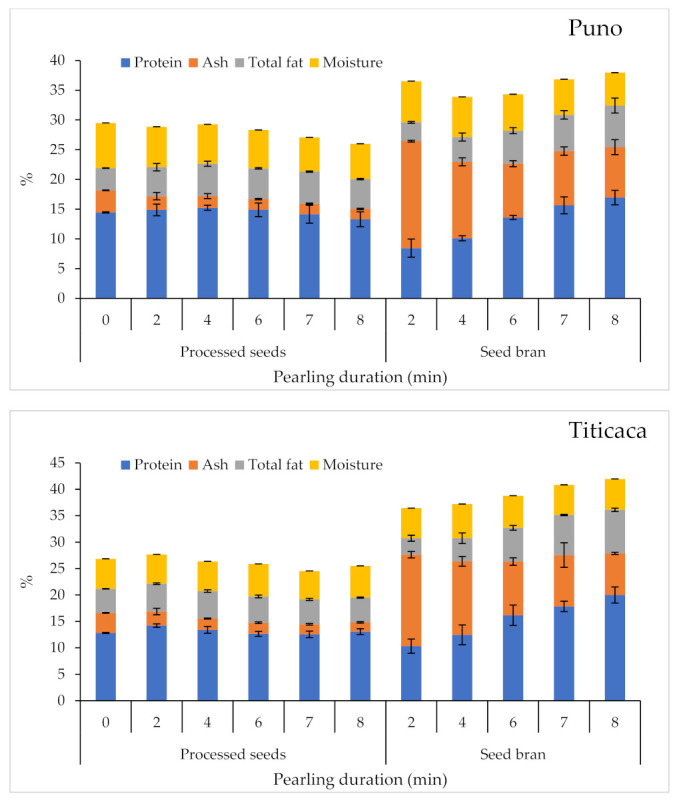

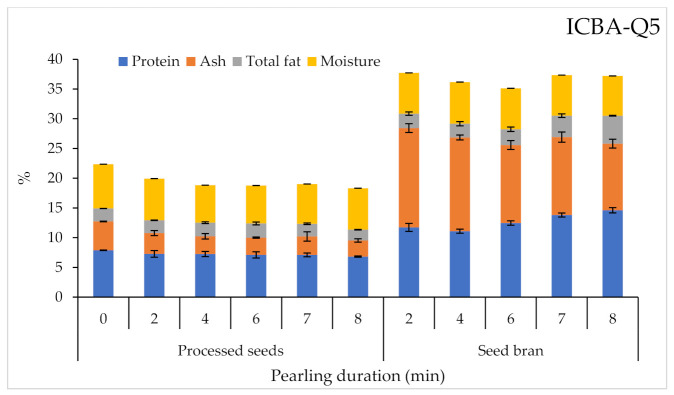
Variations of protein, ash, total fat, and moisture contents in processed seeds and seed bran in response to pearling duration. Error bars indicate the standard deviations.

Figure 4 shows the variations of protein, ash, total fat, and moisture content in processed seeds and seed bran (by-product) in response to pearling duration. Statistical analysis revealed very highly significant differences (*p* < 0.001) between tested varieties in terms of protein, ash, and total fat, but pearling duration affected only seed moisture. Pearling slightly reduced protein, total fat, and moisture contents for all varieties except for Puno, where total fat content slightly increased with the pearling. However, for quinoa bran, the findings are different from the processed seeds: both variety and pearling showed very highly significant differences for most of the parameters. Protein and total fat content increased when pearling duration increased for all tested varieties, whereas ash content decreased with pearling duration. The data indicate that the Puno variety had the highest protein and total fat contents in the seeds; the ICBA-Q5 variety recorded the lowest. Titicaca had the highest bran contents of protein and total fat; ICBA-Q5 had the highest bran content of ash, and the lowest bran content of protein and total fat; and Puno had the lowest bran content of ash.

#### 2.3.2. Macronutrient Content

**Figure 5:**
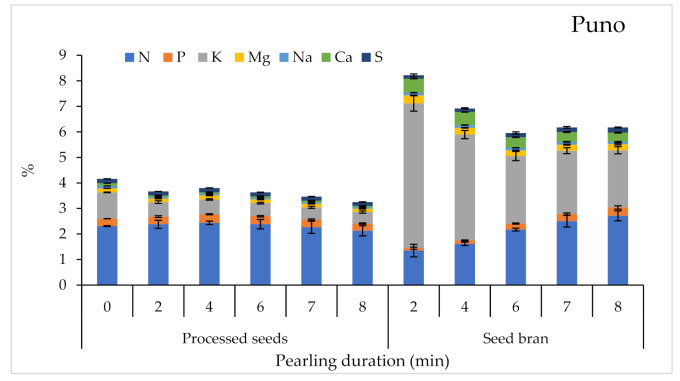

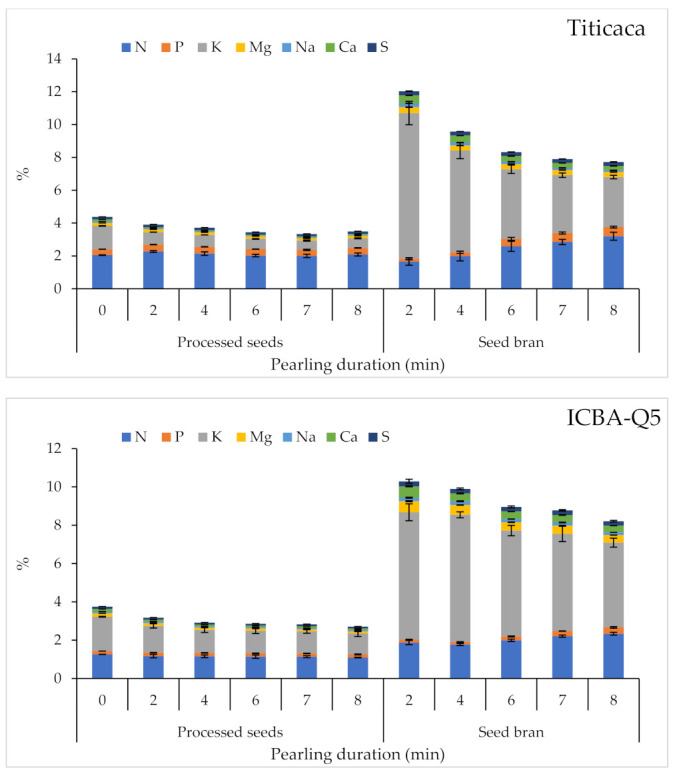
Variations of macronutrient contents in processed seeds and seed bran in response to pearling duration. Error bars indicate the standard deviations.

Macro-nutrient variations in response to pearling duration are presented in Figure 5. Pearling had no significant effect on macronutrient contents in the processed seeds, but it had resulted in very highly significant differences (*p* < 0.001) for most of the macronutrient contents in the bran except Mg and S. The variety effect was obvious and significant for all macronutrients and for both processed seeds and bran. Regarding processed seeds, the highest contents in terms of N, P, and S were recorded for Titicaca; the highest content in terms of K, Mg, and Na for ICBA-Q5; and the highest content in terms of Ca for Puno. Most of the macronutrient contents in seed bran decreased with pearling, except N and P whose contents increased with the pearling.

#### 2.3.3. Micronutrient Contents

**Figure 6:**
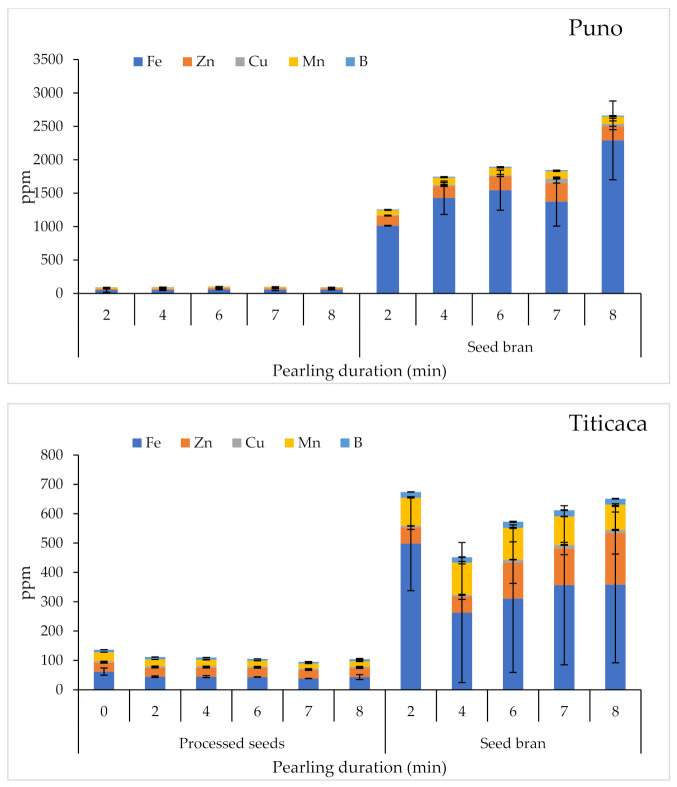

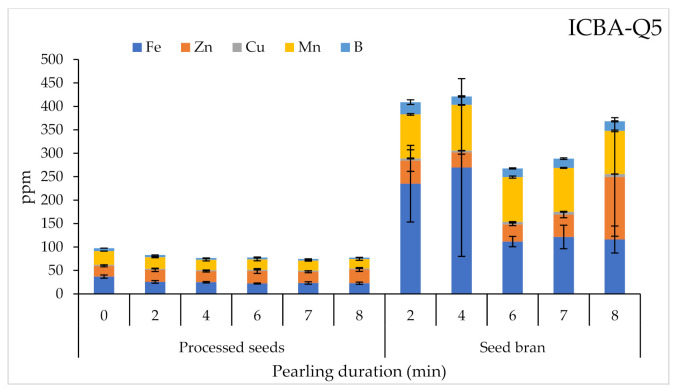
Variations of micronutrient contents in processed seeds and seed bran in response to pearling duration. Error bars indicate the standard deviations.

The effects of pearling on micronutrient contents for the tested quinoa varieties are presented in Figure 6. Statistical analysis showed significant differences (*p* < 0.001) between varieties in terms of Zn, Cu, and Mn contents in processed seeds. Pearling duration had no significant effect on micronutrient contents in processed seeds. Nevertheless, for seed bran, there were very highly significant differences between tested varieties in terms of micronutrients. Pearling had a significant effect only on Zn, which increased with the increased pearling duration.

#### 2.3.4. Saponin Content

**Figure 7:**
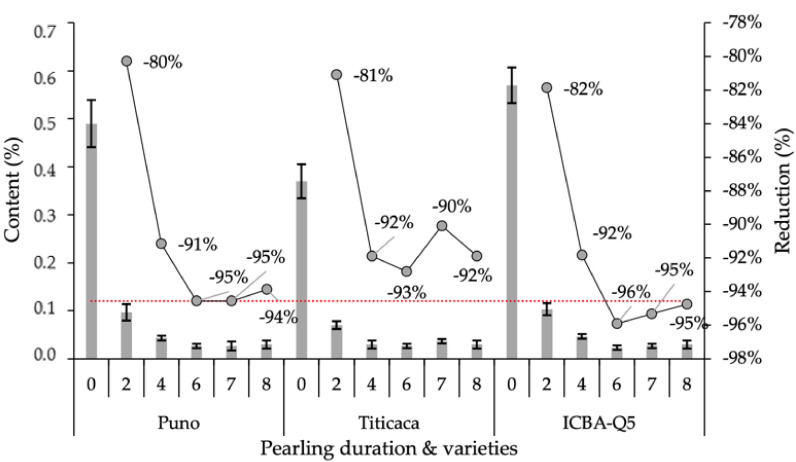
Variations of saponin contents in processed seeds in response to pearling duration. Error bars indicate the standard deviation. Treatments without a common letter are significantly different at *p* < 0.05.

Figure 7 shows the variations of saponin contents for processed seeds in response to several pearling durations. According to statistical analysis, saponin content in processed seeds was affected only by pearling duration; no significant difference was recorded between varieties. However, the interaction between variety and pearling duration factors was significant, which indicates that the tested varieties responded differently to the pearling process. For instance, the ICBA-Q5 variety responded very well to pearling, as it had the highest saponin content in raw seeds (0 min) and the lowest in processed seeds (6 min), and consequently the highest saponin removal.

### 2.4. Pearson Correlation

**Figure 8:**
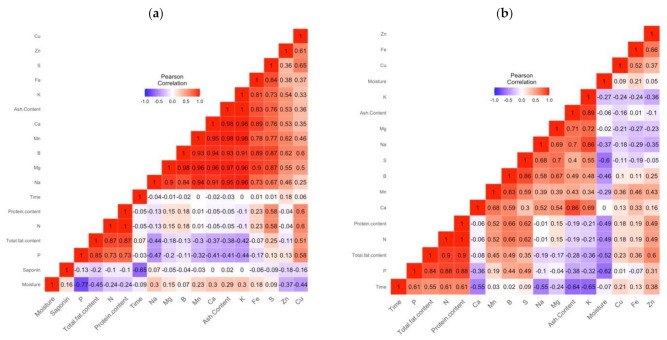
Pearson correlations among all chemical parameters for processed seeds (**a**) and seed brans (**b**).

Pearson’s correlation analysis was conducted for all investigated nutrients in processed seeds and seed bran separately, by correlating all the physicochemical parameters (productibility and growth) with the varieties used. The obtained results are shown in Figure 8.

The analysis revealed a strong positive correlation between groups of parameters G1 (N, protein content) and G2 (Na, Mg, Mn, Ca, ash content, and K). Strong negative correlations were found between moisture and P, saponin, and time. On the other hand, no correlation between time, Mn, K, saponin, Ca, and K was observed (Figure 8a).

For the obtained seed brans (Figure 8b), a strong correlation was found between N, protein content, and total fat. A strong negative correlation was observed between time, ash content, K, moisture, P, and S. No correlation between moisture and Ca was observed.

### 2.5. Principal Component Analysis (PCA)

**Figure 9:**
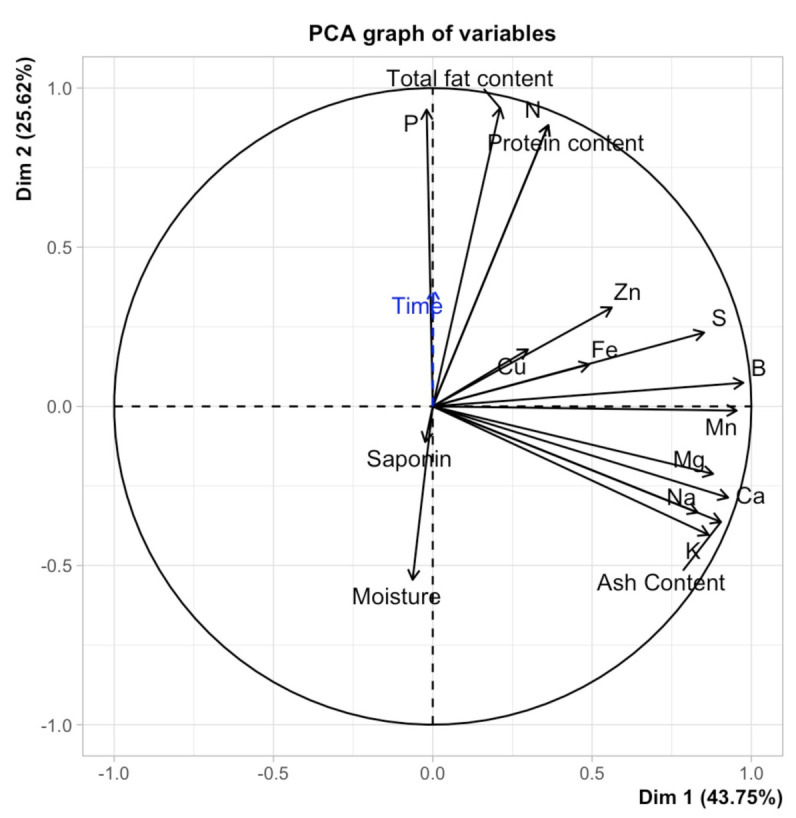
PCA analysis of investigated parameters for processed seeds.

Results of PCA (Figure 9) indicate that the first two components represent over 69% of the variability. PCA axis 1 was largely determined by B, Mn, S, Ca, Na, and K. Among them, B and Ca had the highest positive correlation (r = 0.927–0.975). Variation along PCA axis 2 was highly determined by P, fat content, N, and protein content. P and fat content had the highest positive correlation (r = 0.932–0.937).

The PCA graph of variables showed that the pearling duration was correlated negatively with saponin, moisture, Ca, Na, Mg, and Ca. On the other hand, this key parameter was correlated positively with the rest of the parameters.

## 3. Discussion

Since its introduction to Morocco in the 2000s, Quinoa has been seen as a rustic and stress-tolerant crop with several potentialities to replace cereals and other traditional crops in marginal environments, mainly those affected by drought and salinity. A deep understanding of the nutritional profiling of quinoa and the high amount of saponin reveal the importance of processing in order to remove saponins and produce a palatable seed. Regarding the morphological aspects of quinoa seeds, the quinoa seed is disc-shaped, approximately 2 mm in diameter, and 0.5 mm in thickness. The size of a quinoa seed depends on several factors, such as quinoa cultivar and abiotic conditions (climate, soil, etc.). In fact, the whole quinoa seed is divided into three main structural components: perisperm, embryo, and bran. The bran is defined as the out layer that surrounds the embryo. The embryo entirely covers the perisperm, the innermost layer, as a headband. The bran is eliminated during abrasion (manual or mechanical) because it contains 86% saponins compared to other grain fractions [29]. The embryo and perisperm contain 11% and 3% of saponins, respectively [29].

The special structure of quinoa seeds requires special scarification methods, and the dehulling rates change when milled for the same rate of duration using different equipment [30]. Hence, it is necessary to have a balance between the pearling duration and the nutritional contents of quinoa. For all used varieties, the thousand-seed weight of quinoa seeds decreased with increase pearling duration due to the removal of the external layer of quinoa [31]. Some research works have mentioned that the average diameter can reveal some quinoa characteristics, such as hardness coefficient and saponin content. Otherwise, the higher the hardness coefficient of quinoa, the more saponins are present [32]. The average thousand-seed weight was negatively correlated with the pearling duration. Our finding indicates that after 2 min of mechanical abrasion, a high percentage of saponins were removed.

There are two main processes of saponin removal. The first one is a wet method based on washing. It increases seed moisture, which then requires drying to prevent mold growth, and enhances the seed’s shelf life (while raising the cost of seed processing) [10]. The second method, which is the commonest one, is a dry method based on abrasion that removes the outer bran layer by mechanical scarification. Often, processing operations involve a preliminary washing step following abrasion to further reduce the level of saponins [33]. Nevertheless, for some bitter quinoa varieties that contain higher amounts of saponins, if a wash operation is skipped, the embryo may be damaged as the next outer layer after the bran, which is totally eliminated [34]. Quinoa’s embryo is the richest nutritional source; it contains 57% of the protein, 49% of the fat, 20% of the sugar, 45% of the dietary fiber, and 51% of the ash of the whole grain [29]. Hence, removal techniques that keep the embryo intact preserve the complete nutritional profile of quinoa. Abrasion alone after 30 s reduced saponin levels from 1.19% to 0.45%. However, after an additional 2 min, the saponin level was reduced slowly to 0.21% [33]. Our results showed the same tendency of reducing saponins for the three studied varieties. Two minutes of mechanical abrasion was enough to reduce saponins below 0.12% which is the accepted threshold for quinoa consumption [35]. On the other hand, washing alone augmented the saponin percentage of oleanolic acid, while diminishing phytolaccagenic acid. Although abrasion decreased the percentage of oleanolic acid, it increased the percentage of phytolaccagenic acid [33]. These variations are correlated with the chemical structure of the specific saponin and the location within the bran layer. Furthermore, hydrating the quinoa without manual abrasion increased the saponin levels from 3.3% to 3.6% [32]. The mechanical polishing permitted a reduction of saponins from 2% to 0.4% for Titicaca variety, and from 1.4% to 0.5% for Puno, decreases of 80% and 64% of the initial saponin respectively [36].

The protein contents of the studied quinoa cultivars were equal to 14.4%, 12.8%, and 7.9% (dry weight basis) for Puno, Titicaca, and ICBA-Q5, respectively. Puno and Titicaca’s protein contents were consistent with the protein content (9.5–15.7%, dry weight) reported by Nowak Verena [37]. However, protein content for each variety did not change during the mechanical abrasion. The protein loss increased in terms of seed weight when the pearling duration was higher (after 4 min of mechanical abrasion). The decrease in weight can be explained by the fact that the protruding scutellum of the pericarp of quinoa was scoured away [38,39]. Furthermore, when pearling duration was greater than 6 min, protein loss became greater, since it is primarily present in an embryo, which accounts for 57% of total quinoa protein [37]. Our finding indicates that the embryo and endosperm were milled at this stage of the pearling process.

The fat contents of quinoa cultivars were equal to 3.7%, 4.5%, and 2.2% (dry weight basis) for Puno, Titicaca, and ICBA-Q5, respectively. All contents fit within the range of the total lipid content of quinoa [40]. The increase of total fat after 2 min of mechanical abrasion is explained by the structure and nutritional distribution of quinoa kernels, as 49% of the total fat content of quinoa is present in the embryo, whereas the pericarp contains just 5% of the total fat [29,30]. Moreover, we noticed a decrease in fat content after 6 min of mechanical abrasion, as the bran was removed and the embryo was ground. The obtained results are aligned with another study showing that a dehulling rate of 8.6% can decrease fat content by 7.6% [41].

The ash contents for the three studied varieties fit within the range reported in the literature [40]. The ash content of treated seeds decreased with the increase of the pearling duration. After 2 min of mechanical abrasion, the ash content decreased sharply for all studied varieties. The decrease of ash content is explained by the fact that the pericarp, where most of the minerals are mostly concentrated, was removed. On the other hand, the seed bran obtained from two minutes of mechanical abrasion is highly rich in minerals, and the ash contents were equal to 18.0%, 17.3%, and 16.7% (dry weight basis) for Puno, Titicaca, and ICBA-Q5, respectively. In another study, Ando et al. [29] reported that the ash content was about 9.2% (dry weight basis) in the bran after pearling. That is relatively lower than the content found in this work, which can be explained by the use of different pearling processes and equipment.

A suitable pearling duration is the one that results in saponin content lower than the threshold required from the CODEX standard [35]. Thus, two minutes of mechanical abrasion was the best pearling duration. In this study, we found that K was the most abundant macroelement for Puno, Titicaca, and ICBA-Q5. The remaining macroelements, by abundance, were P, followed by Mg, Ca, S, and then Na. Regarding micronutrients, Fe was the most abundant element with concentrations in raw seeds of 55.2 mg/kg for Puno, 61.8 mg/kg for Titicaca, and 36.81 mg/kg for ICBA-Q5. Our findings in this regard are in agreement with results reported by many other studies where K and P were the dominant macroelements, followed by Mg and then Ca. Fe was the dominant micronutrient [29,36,42]. The quinoa cultivated in Morocco is characterized by higher amounts of K, Ca, and Mg compared to other studies focusing on mineral profiling of quinoa seeds [29,42,43]. Mhada et al. [36] explained that these high contents are mainly due to calcareous soil rich in K, Ca, and other elements.

Our results suggest that protein and mineral contents were not affected by pearling, which indicates that protein and most of minerals are mostly located in the endosperm and embryo, which confirm the finding reported by Prego et al. [44]. According to Konishi et al. [45], the P, K, and Mg are located in embryonic tissue. In particular, P’s origin is attributed to phytic acid, and the origins of Mg and K to phytate. The same authors also reported that K and Ca were present in the pericarp [45]. Furthermore, they found that Ca is scarcely found in embryonic tissues of quinoa seeds. It occurred mostly in the pericarp and seed coat, as well as the boundary between perisperm and embryo. It is associated with carboxyl groups of pectin molecules in the cell wall to form Ca-pectin complexes [44], which confirms the finding of this study that showed decreases in Ca of 58%, 64%, and 14% for Puno, Titicaca, and ICBA-Q5, respectively. Moreover, the decreases in K (56%, 56%, and 16% for Puno, Titicaca, and ICBA-Q5, respectively) can be explained by the fraction located in the pericarp; the K fraction located in the embryo was not eliminated. According to Ando et al. [29], milled quinoa had lower K, Mg, and Ca, and higher P. Nevertheless, the authors also found that Fe, Zn, Cu, and Mn contents remained stable [29]. This disagreement may be explained by some differences in terms of genetic material, since they worked with another variety (quinoa Real) and a lower degree of polishing by the pearling machines used for seed processing [29,36]. According to a previous study conducted with the same pearling machine, the authors found that the pearling process had no effect on macronutrients and protein content, except for Ca, which declined by 28% for Puno variety [10,11]. However, micronutrient contents were significantly reduced. The pearling process resulted in significant losses for most micronutrients—by 60%, 57%, 2%, and 6% for Cu, B, Fe, and Zn, respectively; however, there was no significant reduction in terms of Mn [10,11].

During the pearling process, an important portion of the nutrients is wasted in the produced seed bran; therefore, its valorization is essential. All seed brans generated from the three studied varieties are rich in nutrients compared to the raw seeds. This is explained by the fact that the pericarp has high mineral content, as all produced seed brans contain high ash content. In fact, the research findings demonstrate the richness of quinoa seed brans. However, their valorization will mainly depend on several parameters (saponin contents, microelement contents, and microelement contents), and their usage scope is vast: they can be valorized in food, cosmetic, pharmaceutical, or agronomic industries. Recently, many studies were conducted to valorize saponins in different sectors. For instance, the effect of saponins on pests was studied. Some recent studies suggested the implementation of saponins as an ingredient for the storage of food and grains in order to preserve their sanitary quality. In fact, saponins rupture the internal mucous cells of pest’s intestines by forming complexes with digestive enzymes such as proteases [46]. According to Zegarra [47], the acaricidal activity was evaluated from saponins present in bitter quinoa varieties. The author indicated that the Markjo variety shows bioactivity close to a commercial acaricide product. Furthermore, saponins have a tremendous inhibitory effect on fungi such as botrytis cinerea [48]. Quinoa saponins and polyphenols have high antioxidant effects and free radical scavenging activities, as well as an anti-inflammatory effect [49]. Drugs that contain saponins improve the body’s immune response significantly through the enhancement of absorption and their hemolytic properties. Saponins grabbed worldwide attention due to their anti-carcinogenic characteristics and cholesterol-lowering effects [50]. In food industries, saponins have a positive effect on the physical stabilization of oil/water (O/W) emulsions. Moreover, they contribute to preserving liquids from oxidation, and they can be incorporated as foaming agents in beverage industries [50,51].

## 4. Materials and Methods

### 4.1. Seed Material

Quinoa seeds (*Chenopodium quinoa* Willd) grown in Berrechid, Morocco (2018 harvest) were used in this experiment. Seeds of three varieties of quinoa (Puno, Titicaca, and ICBA-Q5) were used after threshing and winnowing operations to study the effects of several pearling durations on seed nutritional and anti-nutritional properties. Quinoa seeds were not unisized before the pearling process, since it is not important to ensure this additional postharvest operation as well as simulating the real conditions that will be adopted by farmers.

### 4.2. Quinoa Pearling Machine Design

**Figure 10:**
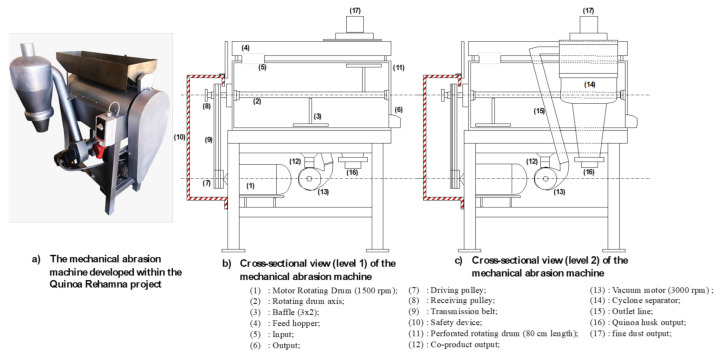
Design of the quinoa pearling machine used in the present experiment.

The pearling machine used in the experiment was locally manufactured by Benrim farm (Figure 10). It is equipped with two motors. The first one is designed to turn a drum with a rotation speed of 750 rpm. The second one is more powerful (3000 rpm) and designed to extract the fine dust produced during the pearling process. The rotating drum is made of an 80 cm long perforated inox steel and has six baffles distributed throughout the drum.

### 4.3. Pearling Treatments and Machine Operating Mode

The machine operates on a semi-industrial scale—a minimal quantity of 8 kg per sample is required. In this trial, five pearling durations were tested (0, 2, 4, 6, 7, and 8 min) for the three selected varieties; 0 min corresponds to raw seeds. Each combination pearling duration x variety was repeated 3 times. For each duration, a 500 g sample was taken, and a quantity of the seed bran recovered for each step was measured. During seed processing, the speed of rotation and friction (seed-seed, seed-drum, and seed-baffles) gradually increased the temperature of the seeds to 40 °C and decreased the moisture from 13% to 10%. After carrying out the pearling process, we collected 99 samples, including 45 processed quinoa samples, 45 seed bran samples, and 9 samples of the untreated raw seeds of the three tested varieties.

### 4.4. Physical Analysis

A precision electronic balance (reading to 0.001 g) was used to determine the 1000 SW by weighing 100 seeds in triplicate and then extrapolating this weight to get 1000 SW [52]. Pictures of seeds were taken using optical microscopy Nikon Eclipse Lv100nd.

### 4.5. Chemical Analysis

After processing, quinoa samples were ground to a fine powder using FOSS CT 293 Cyclotec grinder with a range of screens of 0.3 mm.

The moisture content was measured by drying 100 g of the sample at 105 °C for 48 h according to ICC Standard 110/1. The protein, fat, and ash contents were determined according to AOAC procedures [53].

For the evaluation of protein content, a macro-Kjeldahl method (N × 6.25) was used. The fat content was determined by Soxhlet method, using hexane solvent for the extraction for 4 h [54].

The ash content was determined according to the AOAC 923.03 standard. Micro-nutrients were determined after sample mineralization. Representative samples (0.25 g) were digested with 7.5 mL of nitric acid (HNO3) in a DigiPrep system for two hours at 100 °C. After digestion, the solutions were filtered through 45 µm filters, and the filtrates were diluted to 50 mL with deionized water and acidified (2% HNO3) in order to undergo the analysis by ICP-OES using Agilent technologies 5110 ICP-OES [10].

All measurements were performed in triplicate.

### 4.6. Saponin Determination by Liebermann–Buchard Colorimetric Assay

The protocol allows the determination of triterpene saponins from quinoa seeds. The principle of the assay is based on the reaction between the Liebermann–Burchard (LB) reagent and the triterpene molecules by developing a color [55].

#### 4.6.1. Determining the Wavelength for the Dosage

A series of absorbance readings were taken in different wavelengths to get the wavelength corresponding to the maximum absorbance. We found that the maximal absorbance corresponds to 528 nm.

#### 4.6.2. Dilutions for Range Preparation

According to Table 3, a series of standard solutions (0.2, 0.3, 0.5, and 0.8 mL) were taken, and the mixed solutions were prepared by the method described above for measuring absorbance values.

#### 4.6.3. Saponin Extraction

Five grams of the defatted quinoa powder (Soxhlet extraction by using hexane solvent for 4 h and repeated three times) was taken in 50 mL of ethanol. The mixture was then shaken vigorously for 30 min to extract total saponins. Then, the mixture was filtered using a filter paper (*Fironi standard—Plisse*). The filtrate was collected and topped up to 50 mL with ethanol.

#### 4.6.4. Development of the color

**Figure 11:**
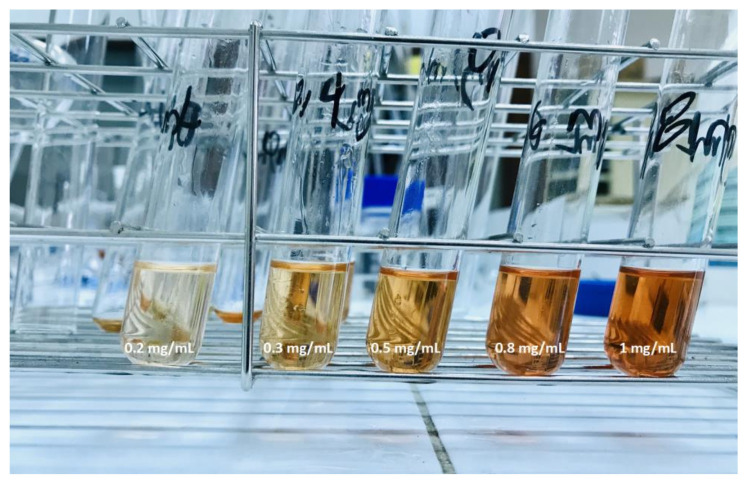
Different concentrations of the colored solutions.

The saponin content determination was carried out according to the method applied by Irigoyen et al. [55], by adding 1 mL of the diluted solution to 3.5 mL of the Lieberman-Buchard reagent (16.7% acetic anhydride in concentrated sulfuric acid). The solution was vortexed and stored in the dark for 30 min at room temperature. The absorbance of the solution was measured at 528 nm in a spectrophotometer (Agilent 8453 Spectrophotometer, Los Angeles, CA, USA). Figure 11 shows the development of the color for each dilution.

#### 4.6.5. Calibration Curve Preparation

Regarding the preparation of the stock solution of the standard, oleanolic acid (97%) (Sigma-Aldrich, St. Louis, MO, USA) was used as the pure standard for triterpene saponin; 10 mg of OA was dissolved in 10 mL of pure ethanol to obtain a stock solution concentration of 1 mg/mL [56].

**Figure 12:**
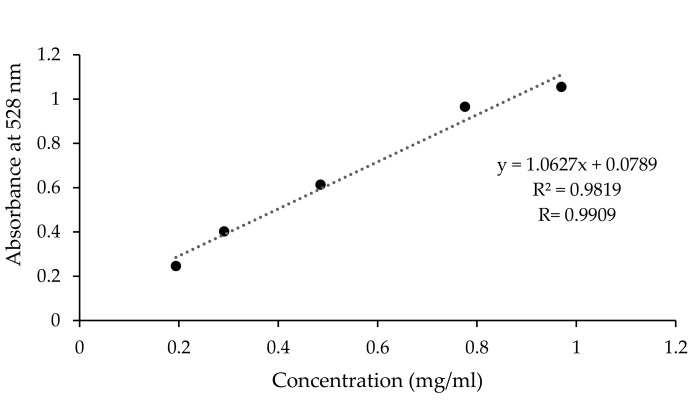
Calibration curve of oleanolic acid at 528 nm.

The range of absorbance obtained after dilutions was between 0.2 and 1 mg/mL which corresponds to an absorption of 0.246 to 1.055 (Figure 12). Beer–Lambert’s law is applicable in the straight part of the trace, and the range of linearity is between 0.2 and 0.8 absorbance units.

From the calibration curve, X is calculated as the following:

(1) $$x\left(\frac{mg}{ml}\right)=\frac{y-0.0789}{1.0627}$$

The concentration of saponins is calculated according to the following equation:

(2) $$Concentration\;\left(\frac{g}{100g}DM\right)=\frac{x\left(\frac{mg}{ml}\right)\times V_{D}\times 10^{-3}\times 100\times 100}{\left(1+x\left(Fat\right)\right)\times Sample\;weight\;\left(g\right)\times \left(100-Moisture\right)}$$

### 4.7. Statistical Analysis

Statistical analysis was carried out using R version 3.6.2 software. A two-way analysis of variance (ANOVA) was used to assess the effects of the pearling process on each variety (physical and chemical parameters). The level of significance was set to *p* < 0.05.

Pearson’s correlation coefficient was evaluated according to the linear correlations between any two parameters. If the obtained coefficient was −1 or +1, there was a perfect negative or positive linear relationship, respectively. The value of 0 denotes no correlation between the two variables. Generally, a correlation could be statistically significant at a 95% confidence interval (*p* < 0.05).

Principal component analysis (PCA) was used to investigate the potential correlation matrix between all the measured parameters and the pearling time, as well as the data groupings after the pearling treatment.

## 5. Conclusions and Recommendations

One of the limiting factors facing quinoa valorization and consumption in Morocco and other countries is the seed content of saponin. Furthermore, traditional ways of removing saponins using manual abrasion and washing require intensive labor and thus increase production costs. Therefore, developing mechanical dry pearling tools for saponin removal will solve two interlinked challenges—reducing the processing costs and saving water, thereby increasing farmer’s income.

In our study, we tested three quinoa varieties grown in Morocco and polished with a locally manufactured machine. As most cleaning and saponin removal is done under wet or humid methods, our method by dry abrasion had advantageous results, which can be beneficial for farmers and processors.

The findings of this study clearly indicate that two-minute pearling was enough to remove saponins and keep their concentration below the consumption threshold, while at the same time preserving both physical and nutritional quality of the seeds. This study highlights the potentialities of the locally manufactured machine to be used in other regions in Morocco or in other countries, especially as it is a low-cost tool and could be easily operated by smallholder farmers.

## Figures and Tables

**Table 1:** Results of ANOVA (analysis of variance) for processed seeds and seed bran—the effects of pearling on nutritional and saponin contents of quinoa seeds.

Product	Factor	1000 SW	PR	A	TF	M	Macro-Elements							Micro-Elements						SP
							N	P	K	Mg	Na	Ca	S	Fe		Zn	Cu	Mn	B	
Processed seeds	V	***	***	***	***	***	***	***	***	NS	***	***	NS	NS		***	*	**	NS	NS
	PD	***	NS	NS	NS	***	NS	NS	NS	NS	NS	NS	NS	NS		NS	NS	NS	NS	***
	V × PD	NS	NS	NS	NS	*	NS	NS	NS	NS	NS	NS	NS	NS		NS	NS	NS	NS	*
	*p*-value	0.223	0.439	0.855960	0.133	0.022630	0.439	0.358	0.904432	0.9115	0.939080	0.968891	0.889	0.961		0.145	0.7194	0.73797	0.9343	0.0469
Seed bran	V	-	***	NS	***	***	***	***	**	*	***	***	***	***		***	**	***	**	-
	PD	-	***	***	***	***	***	***	***	NS	***	***	NS	NS		***	NS	NS	NS	-
	V × PD	-	***	NS	***	**	***	***	*	NS	**	NS	NS	*		NS	NS	NS	NS	-
	*p*-value		6.09 × 10^−5^	0.164	1.01 × 10^−7^	0.001727	6.09 × 10^−5^	5.82 × 10^−6^	0.01169	0.9239	0.00407	0.737929	0.095	0.0317	0.568709		0.25700	0.097694	0.1639	-

V: variety; PD: pearling duration; NS: non significant; -: no data available; *: significant difference; **: highly Significant; ***: very highly significant; SW: seed weight; PR: protein; A: ash; TF: total fat; M: moisture; N: nitrogen; P: phosphorus; K: potassium; Mg: magnesium; Na: sodium; Ca: calcium; S: sulfur; Fe: iron; Zn: zinc; Cu: copper; Mn: manganese; B: boron; SP: saponin.

**Table 2:** Results of Tukey’s HSD post hoc test for both processed seeds and seed bran—the effects of pearling on the nutritional content of quinoa seeds.

Variety/Product	PD	Proximate Analysis				Macro-Nutrients								Micro-Nutrients				
		PR	A	TF	M	N	P	K	Mg	Na	Ca	S	Fe		Zn	Cu	Mn	B
**Puno**																		
Processed seeds	0	a	a	b	a	a	a	a	a	a	a	a	a		b	a	a	a
	2	a	b	ab	ab	a	a	b	ab	ab	b	a	a		ab	a	bc	ab
	4	a	b	a	b	a	a	b	ab	b	b	a	a		ab	a	ab	ab
	6	a	b	a	bc	a	a	b	ab	b	b	a	a		ab	a	bc	ab
	7	a	b	a	c	a	a	b	ab	ab	b	a	a		ab	a	bc	ab
	8	a	b	ab	bc	a	a	b	b	ab	b	a	a		ab	a	c	b
Seed bran	2	d	a	c	a	c	a	a	a	a	a	c	a		a	a	b	a
	4	cd	b	c	a	c	a	b	ab	a	a	bc	a		a	a	ab	b
	6	bc	c	b	a	b	a	c	b	a	b	ab	a		a	a	a	b
	7	ab	c	ab	a	ab	b	c	b	a	b	a	a		a	a	ab	b
	8	a	c	a	a	a	b	c	b	a	b	a	a		a	a	ab	b
ICBA-Q5																		
Processed seeds	0	a	a	a	a	a	a	a	a	a	a	a	a		a	a	a	a
	2	a	a	a	ab	a	a	a	a	a	a	a	a		a	a	a	a
	4	a	a	a	ab	a	a	a	a	a	a	a	a		a	a	a	a
	6	a	a	a	ab	a	a	a	a	a	a	a	a		a	a	a	a
	7	a	a	a	b	a	a	a	a	a	a	a	a		a	a	a	a
	8	a	a	a	b	a	a	a	a	a	a	a	a		a	a	a	a
Seed bran	2	a	a	a	a	a	a	a	a	a	a	a	a		a	a	a	a
	4	a	a	a	a	a	a	a	a	ab	a	a	a		a	a	a	a
	6	a	a	a	a	a	a	a	a	b	a	a	a		a	a	a	a
	7	a	a	a	a	a	a	a	a	ab	a	a	a		a	a	a	a
	8	a	a	a	a	a	a	a	a	ab	a	a	a		a	a	a	a
Titicaca																		
Processed seeds	0	a	Ash	b	a	a	a	a	a	a	a	a	a		b	a	a	a
	2	a	b	ab	ab	a	a	b	ab	ab	b	a	a		ab	a	bc	ab
	4	a	b	ab	b	a	a	b	ab	b	b	a	a		ab	a	ab	ab
	6	a	b	ab	bc	a	a	b	ab	b	b	a	a		ab	a	bc	ab
	7	a	b	ab	c	a	a	b	ab	ab	b	a	a		ab	a	bc	ab
	8	a	b	ab	bc	a	a	b	b	ab	b	a	a		ab	a	c	b
Seed bran	2	d	a	c	a	d	b	a	a	a	a	a	a		b	c	ab	a
	4	cd	b	c	a	cd	b	b	b	b	b	a	a		b	bc	a	a
	6	bc	c	b	a	bc	a	c	b	bc	c	a	a		ab	ab	ab	a
	7	ab	c	ab	a	ab	a	c	b	c	d	a	a		ab	a	ab	a
	8	a	c	a	a	a	a	c	b	c	e	a	a		a	a	b	a

PD: pearling duration; PR: protein; A: ash; TF: total fat; M: moisture; N: nitrogen; P: phosphorus; K: potassium; Mg: magnesium; Na: sodium; Ca: calcium; S: sulfur; Fe: iron; Zn: zinc; Cu: copper; Mn: manganese; B: boron. Treatments without a common letter are significantly different at *p* < 0.05.

**Table 3:** Dilution series for the standard curve determination.

0.8 mg/mL	$Fd=\frac{Vf}{Vi\;}=\frac{1\left(\frac{mg}{ml}\right)}{0.8\left(\frac{mg}{ml}\right)}=1,3$	1 mL of the stock solution and 0.3 mL of ethanol.	1 mL of the diluted solution is taken.
0.5 mg/mL	$Fd=\frac{Vf}{Vi\;}=\frac{1\left(\frac{mg}{ml}\right)}{0.5\left(\frac{mg}{ml}\right)}=2$	0.5 mL of the stock solution and 0.5 mL of ethanol.	1 mL of the diluted solution is taken.
0.3 mg/mL	$Fd=\frac{Vf}{Vi\;}=\frac{1\left(\frac{mg}{ml}\right)}{0.3\left(\frac{mg}{ml}\right)}=3.3$	0.3 mL of the stock solution and 0.7 mL of ethanol.	1 mL of the diluted solution is taken.
0.2 mg/mL	$Fd=\frac{Vf}{Vi\;}=\frac{1\left(\frac{mg}{ml}\right)}{0.2\left(\frac{mg}{ml}\right)}=5$	0.5 of the stock solution and 4.5 mL of ethanol.	1 mL of the diluted solution is taken.

## Data Availability

The data generated within this work are open access and available to be shared with interested persons according to signed agreement with IDRC.
